# A case report of a family with *MYH9* gene mutation-related disease in an ethnic minority group and literature review

**DOI:** 10.1186/s12920-026-02347-0

**Published:** 2026-03-28

**Authors:** Xia Yan, Zhenzhen Li, Shaojun Huang, Xiaoxia Yang, Ming Chang, Zhengjiang Cheng, Lan-Ting Zhou

**Affiliations:** 1 Department of Medical Laboratory, Xiangyang Central Hospital, Affiliated Hospital of Hubei University of Arts and Science, Xiangyang, China; 2 Department of Obstetrics and Gynecology, Xiangyang Central Hospital, Affiliated Hospital of Hubei University of Arts and Science, Xiangyang, China; 3 Hubei Provincial Clinical Research Center for Cervical Lesions, Xiangyang, China

**Keywords:** *MYH9*, May-Hegglin anomaly, Blood smear, Whole-exome sequencing (WES), Sanger sequencing

## Abstract

**Background:**

May-Hegglin anomaly, a rare autosomal dominant disorder caused by *MYH9* mutations, is characterized by the classic "triad" of thrombocytopenia, giant platelets, and granulocyte cytoplasmic inclusion bodies; some patients also present non-hematological symptoms.

**Results:**

We reported a family of *MYH9*-related disease. The proband had microscopic hematuria, proteinuria, and thrombocytopenia on physical examination; blood smear re-examination showed giant platelets and Döhle-like bodies. His medical history included childhood epistaxis; his father had unexplained thrombocytopenia/proteinuria, and his younger brother had epistaxis/purpura/thrombocytopenia. Whole-exome sequencing (validated by Sanger sequencing) confirmed the diagnosis.

**Conclusions:**

Diagnosis of *MYH9*-related diseases depends on combined laboratory morphology and molecular biology. Routine blood tests and smear microscopy (identifying abnormal giant platelets/Döhle-like bodies) provide initial screening clues, while gene sequencing enables accurate diagnosis and pathogenesis clarification, forming a complete screening-to-confirmation diagnostic pathway.

**Supplementary Information:**

The online version contains supplementary material available at 10.1186/s12920-026-02347-0.

## Introduction

*MYH9*-related diseases (MYH9-RD) are rare autosomal dominant disorders caused by pathogenic mutations in the *MYH9* gene (encodes non-muscle myosin heavy chain IIA, NMMHC-IIA). Epidemiological data show an incidence of ~1–9 cases per million, with sporadic cases reported across global ethnic groups [1]. MYH9-RD exhibits dual clinical features involving both hematological and non-hematological systems. Typical hematological manifestations include early-onset giant platelets, thrombocytopenia, and the formation of Döhle-like bodies in granulocyte cytoplasm, which further lead to bleeding tendencies of varying severity.

MYH9-RD encompass four main subtypes, with core differences focusing on clinical manifestations and gene mutation loci: May-Hegglin anomaly (MHA) is characterized by thrombocytopenia, giant platelets, and Döhle-like bodies, typically without significant multi-organ involvement [2]. Sebastian syndrome (SBS) shares similar clinical features with MHA, but its Döhle-like bodies are smaller and irregular in shape [3]. Fechtner syndrome (FCS) presents with hematological manifestations, and is often complicated by progressive hearing impairment, cataracts, and nephropathy [4]. Epstein syndrome is characterized by macrothrombocytopenia in the absence of neutrophil inclusion bodies, accompanied by deafness and renal failure [5].

In the relevant literature, MYH9-RD has been defined as a syndrome characterized by macrothrombocytopenia accompanied by Döhle-like bodies, with or without concurrent nephritis and sensorineural hearing loss. Among these, patients presenting with typical manifestations of chronic nephritis such as hematuria and proteinuria are mostly classified into the Fechtner syndrome subtype [6–8].

Given the heterogeneity of its clinical phenotypes and its low incidence, MYH9-RD are highly prone to misdiagnosis in clinical practice as common thrombocytopenic disorders such as immune thrombocytopenia or thrombocytopenic purpura. Therefore, there is an urgent need to enhance clinicians' awareness of this disease and their ability in differential diagnosis.

## Case presentation

**Fig. 1:**
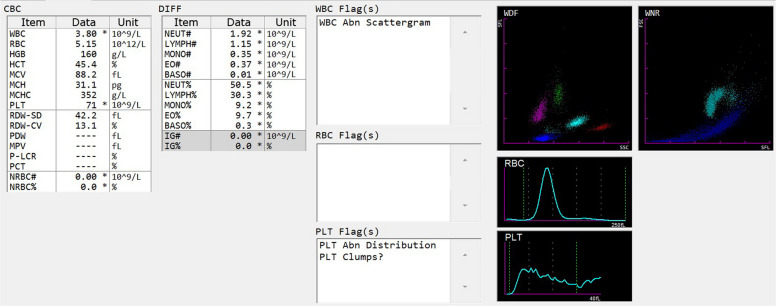
Raw results of the proband’s Complete Blood Count (CBC)

**Fig. 2:**
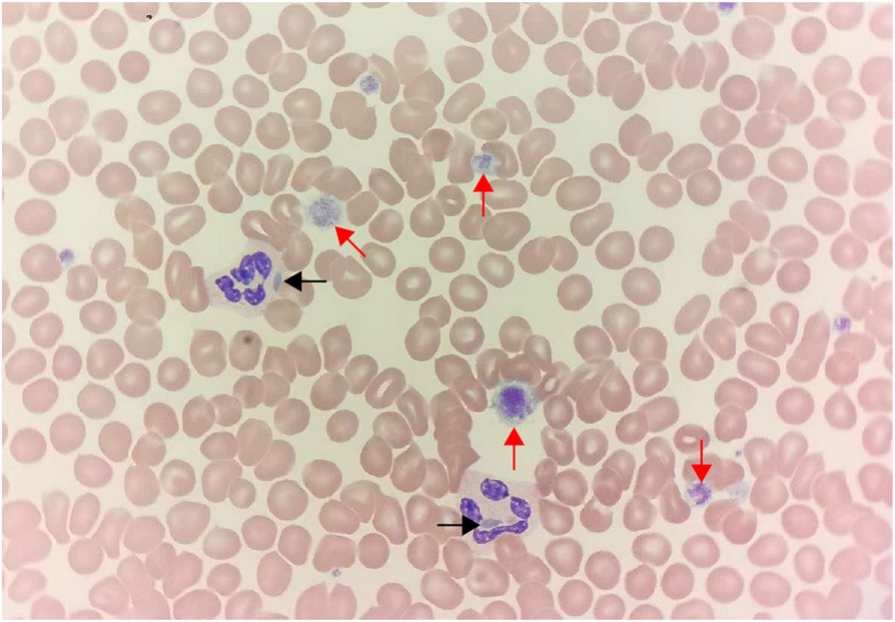
Blood smear of the proband. Note: The black arrow points to Döhle-like bodies, and the red arrow points to giant platelets. Oil immersion lens magnification at 100×

We report a family with May-Hegglin anomaly from the Enshi city, Hubei Province, in which 3 members were diagnosed with MYH9-RD. The proband was a 22-year-old male patient who presented for medical evaluation due to abnormal urinalysis and blood routine findings detected during a physical examination. Urinalysis showed occult blood (2+) and proteinuria (3+), with 8–15 red blood cells per high-power field (RBC/HPF) on urine sediment microscopy (Table 1). Blood routine examination revealed a white blood cell count (WBC) of 3.80 × 10⁹/L, red blood cell count (RBC) of 5.15 × 10^12^/L, hemoglobin (Hb) of 160 g/L, and platelet count (PLT) of 71 × 10⁹/L (Fig. 1). The hematology analyzer alerted to "abnormal white blood cell scattergram, abnormal platelet histogram, and platelet aggregation" (Fig. 1). A review of the blood smear showed that giant platelets accounted for 52% (round/oval in shape, with a volume close to that of red blood cells). Additionally, 2–5 μm spindle-shaped/fusiform Döhle-like bodies (with clear boundaries, mostly distributed at the cell margin) were observed in the cytoplasm of neutrophils, eosinophils, and a small number of monocytes (Fig. 2). Given the proband’s history of recurrent epistaxis in childhood, May-Hegglin anomaly was highly suspected.

**Table 1:** Relevant test results of the pedigree

Patient	Gender	Age (Years)	Clinical Symptoms	Detection Items	Platelet Count	Blood Smear		Urinalysis Analysis			Biochemical Results				
				Test Items Names	Platelet Count	May—Hegglin Bodies	Large Platelet Ratio	BLD	PRO	RBC (/HPF)	Cr	BUN	ALT	AST	GGT
				Unit	(× 10⁹)	-	%	-	-	(/HPF)	(μmol/L)	(μmol/L)	(U/L)	(U/L)	(U/L)
				Reference Value	125—350	-	13—43	-	-	0—13	57—97	3.1—8.0	9—50	15—40	10—60
				Detection Method	Impedance Method	Microscopic Examination	Calculation Method	Dry Chemistry Method	Dry Chemistry Method	Flow Imaging Method	Sarcosine Oxidase Method	Urease—Glutamate Dehydrogenase Method	Rate Method	Rate Method	Rate Method
Proband(Before Treatment)	Male	22	Epistaxis	Test Result	71↓	Present	52↑	2 + ↑	3 + ↑	8—15	74	4.2	30	25	42
Proband(After Treatment)	Male	22	Epistaxis	Test Result	-	-	-	1 + ↑	1 + ↑	5	46	3.5	30	25	42
Father	Male	45	None	Test Result	54↓	Present	50↑	1 + ↑	2 + ↑	0—5	156↑	8.12↑	28	20	22
Mother	Female	45	None	Test Result	231	Absent	2↓	-	-	0	56	4.09	20	12	14
Younger Brother	Male	12	Ecchymosis/Epistaxis	Test Result	30↓	Present	44↑	-	-	0	57	3.22	31	64↑	12

**Fig. 3:**
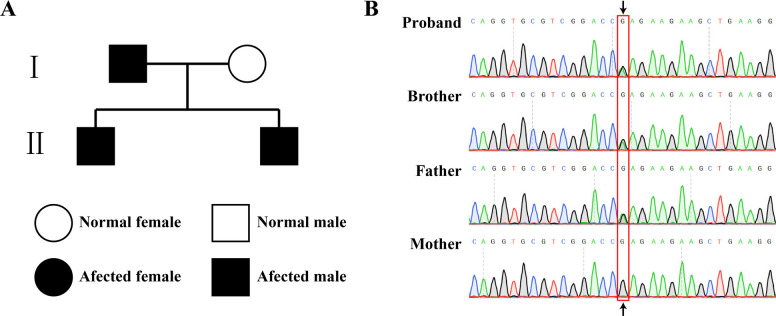
Genetic pedigree and sanger sequencing diagram. **A** the family pedigree. **B** Sanger sequencing chromatogram of *MYH9* gene in this family

Family history investigation revealed that the proband’s father had unexplained thrombocytopenia and proteinuria. His 12-year-old younger brother presented with epistaxis and was found to have a platelet count of 25 × 10⁹/L. He was previously diagnosed with immune thrombocytopenia (ITP) at another hospital, and despite treatment with intravenous immunoglobulin and methylprednisolone, his platelet count only transiently increased to 158 × 10⁹/L before rapidly dropping back to 30 × 10⁹/L, indicating treatment resistance. With informed consent, the family was followed up, and blood test results of 4 family members showed obvious genetic correlation characteristics: the proband, his father, and his younger brother all had platelet counts below the reference range, accompanied by May-Hegglin anomaly and abnormal proportions of large platelets, consistent with the manifestations of autosomal dominant platelet disorders. In contrast, the mother’s platelet count and blood smear indices were normal. Regarding urinalysis, the proband and his father had abnormalities in occult blood, protein, and red blood cells, suggesting renal/urinary system damage. The father also had elevated creatinine and urea nitrogen levels, indicating impaired renal function, while the mother and younger brother had normal urinalysis results. In terms of biochemical enzymology, the younger brother had an elevated aspartate transaminase (AST) level, requiring attention to potential hepatocellular injury; other family members (except the father, whose AST, alanine transaminase [ALT], and gamma-glutamyl transferase [GGT] were normal) had normal liver enzyme indices (Table 1). Auxiliary examinations, including B-ultrasound of the liver, gallbladder, pancreas, spleen, and kidneys, as well as hearing and ophthalmic assessments, showed no abnormalities. Segregation analysis of the family pedigree supported an autosomal dominant inheritance pattern (Fig. 3). Given the fluctuating disease course and progressive symptoms, whole-exome sequencing (WES) was performed using the MGISEQ-2000 platform for all family members to identify the etiological cause and determine the parental origin of the variant, with validation by Sanger sequencing on an AB 3500 genetic analyzer. Results showed that the proband, his father, and his younger brother all carried a heterozygous missense mutation in the *MYH9* gene (NM_002473.4:exon39, c.5521G > A, p.Glu1841Lys), while his mother had the wild-type allele. According to the ACMG criteria, this variant was classified as pathogenic, with supporting evidence including PS4, PS3, PP4, PP2, PP1_Strong, PM6_Strong, and PM2 (evidence items: PS4 + PS3 + PP4 + PP2 + PP1_Strong + PM6_Strong + PM2) (Table 2 and Fig. 3).

**Table 2:** Whole—exome sequencing detection results (Trio)

Chromosomal Position	Gene	cHGVS	pHGVS	Gene Sub—region	Functional Mutation	Match Rate	Heterozygosity ratio (Depth BF) (Proband/Father/Mother/Younger Brother)	Disease Information
chr22:36,680,520	*MYH9*	c.5521G > A	p.Glu1841Lys	EX39	Missense	63%	Proband: Het 0.45 256	AD:May—Hegglin abnormality
							Father: Het 0.4 218	AD:Autosomal dominant deafness type 17
							Mother: NA NA NA	UNK:Prenatal phenotype abnormality

In terms of treatment, the patient was initially treated with herbal remedies due to 3+ proteinuria and 2+ occult blood. However, the therapeutic effect was unsatisfactory after 2 months. Based on the references [9] and genetic testing results, the treatment was adjusted to an angiotensin-converting enzyme inhibitor (ACEI), specifically benazepril hydrochloride (5mg/day, Novartis Pharma Beijing Co., Ltd., NDC: H20000292). After 6 months of treatment, the proteinuria decreased to 1+, and the serum creatinine level was 46μmol/L (Table 1), with no adverse reactions such as hypotension. The proband is currently undergoing regular follow-up. His father and younger brother have not received treatment for the time being but require regular disease monitoring.

## Discussion and conclusions

Patients in this pedigree exhibited the classic hematological triad of MYH9-RD, characterized by thrombocytopenia, giant platelets, and neutrophilic cytoplasmic inclusions. Clinically, the proband and his father presented with mild bleeding tendencies (e.g., mucocutaneous hemorrhage) and renal involvement, manifested as hematuria, proteinuria, and mild elevation of serum creatinine. Currently, no ocular or auditory dysfunction, nor hepatic enzyme elevation, has been detected in these two individuals. In contrast, his younger brother has not yet developed renal impairment. Notably, the proband experienced mild bleeding symptoms during childhood, which gradually resolved with advancing age—this observation highlights the marked clinical phenotypic heterogeneity of the disease. Even within the same pedigree harboring the identical amino acid substitution (the terminal p.Glu1841Lys mutation in the *MYH9* gene) [10], distinct patterns of organ involvement can emerge. This finding supports the hypothesis that the four subtypes of MYH9-RD may represent dynamic pathological stages of progression, influenced by factors such as age, environment, and gender [11, 12]. Although this mutation was initially reported to have relatively weak pathogenicity, accumulating clinical evidence has confirmed its association with varying degrees of organ damage [13, 14]. It was reported a 10-year-old girl diagnosed with MYH9-RD based on cytomorphological findings, a positive family history (maternal thrombocytopenia, with mother's hearing loss and grandmother's renal failure), and identification of the *MYH9* p.E1841K mutation [15].

Currently, reports on this category of diseases remain predominantly case-based, and no definitive statistical data has been established regarding the proportion of organ system involvement or the incidence of life-threatening complications. These warrants heightened attention from clinicians across multiple disciplines for differential diagnosis. For patients with MYH9-RD, it is recommended to establish a multidisciplinary follow-up system involving hematology, nephrology, and otolaryngology departments. Regular monitoring of renal function and hearing should be conducted to enable early intervention for complications. Thrombopoietin (TPO) analogs are a key exploratory direction for the symptomatic treatment of thrombocytopenia in MYH9-RD patients. Recombinant human TPO raised platelet counts and reduced bleeding risk, laying a foundation for clinical use [16]. TPO analogs (e.g., romiplostim) stably maintained platelets, reduced bleeding recurrence long-term, and had no severe adverse reactions [17]. Low-dose TPO analogs were effective and safe for pediatric MYH9-RD patients [18]. TPO response may correlate with *MYH9* mutation type, requiring pre-treatment genetic evaluation [19]. However, regular monitoring of complete blood counts and bone marrow morphological indicators is necessary to ensure safety treatment. In contrast, angiotensin-converting enzyme inhibitors (ACEIs) and angiotensin II receptor blockers (ARBs) may reduce proteinuria and delay the progression of renal dysfunction [7, 8, 20, 21]. A rigorous evaluation of medication selection is required for patients with MYH9-RD [22]. The younger brother of the proband was misdiagnosed with idiopathic thrombocytopenic purpura (ITP) at another hospital due to thrombocytopenia, resulting in ineffective treatment. The primary cause of this misdiagnosis was the laboratory staff’s neglect of morphological differentiation of blue cytoplasmic inclusions in neutrophils: The characteristic May-Hegglin anomaly in MYH9-RD are well-defined, fusiform or spindle-shaped structures that persist in neutrophils, eosinophils, and a small number of monocytes. In contrast, infection-associated Döhle-like bodies are only observed at the periphery of neutrophils, presenting as round, ill-defined structures, often accompanied by infection-related signs such as toxic granulation, and they resolve with the control of inflammation [23].

In reviewing the diagnostic process of this case, the identification of the "triad" through peripheral blood smear microscopy emerged as a critical step. Prominent features include May-Hegglin anomaly in neutrophils and giant platelets visible under low-power microscopy. The Guidelines for Standardization of Blood Cell Analysis Reports explicitly state that when abnormal Döhle-like bodies are accompanied by macrothrombocytopenic thrombocytopenia, a high suspicion for MYH9-RD should be raised, and genetic testing is recommended [24]. It is important to note that some patients may lack detectable inclusions in their peripheral blood smears; thus, genetic diagnosis serves as a necessary supplement to rule out misdiagnosis.

In summary, diagnosis of MYH9-RD requires integrating morphological laboratory findings and molecular biology. Routine blood tests and peripheral blood smear microscopy are key screening tools; laboratory personnel should enhance morphological review after alerts from automated hematology analyzers. Standardizing blood cell reports (e.g., documenting inclusion morphology) improves MYH9-RD detection, and early accurate diagnosis enhances patient prognosis while reducing the medical burden of end-stage organ damage.

## Supplementary Information

Supplementary Material 1.

Supplementary Material 2.

Supplementary Material 3.

## Data Availability

The datasets generated and/or analysed during the current study are available in the Genome Sequence Archive (GSA) repository, [https://ngdc.cncb.ac.cn/gsa-human/submit/hra/subHRA019263/finishedOverview].

## Abbreviations

*MYH9*: Nonmuscle myosin heavy chain 9 gene
MYH9-RD: *MYH9*-Related disease
WES: Whole-exome sequencing
