# Supplementary material for: A case report of a family with MYH9 gene mutation-related disease in an ethnic minority group and literature review
Source: BMC Med Genomics. 2026 Mar 28;19:73. doi: 10.1186/s12920-026-02347-0 (PMC13151366; doi:10.1186/s12920-026-02347-0)
Supplement: Supplementary file 2 — Supplementary Material 2. [file 12920_2026_2347_MOESM2_ESM.pdf]

Patients	SampleID	#Chr	Start	Stop	Ref	Call	Gene Symbol	Transcript	cHGVS	pHGVS	ExIn_ID	Function	Zygosity	A.Depth	A.Ratio
Proband	23S1031106	chr22	36680519	36680520	C	T	MYH9	NM_002473.4	c.5521G>A	p.E1841K | p.Glu1841Lys	EX39	missense	Het	256	0.45
Brother	23S1031107	chr22	36680519	36680520	C	T	MYH9	NM_002473.4	c.5521G>A	p.E1841K | p.Glu1841Lys	EX39	missense	Het	260	0.55
Father	23S1031108	chr22	36680519	36680520	C	T	MYH9	NM_002473.4	c.5521G>A	p.E1841K | p.Glu1841Lys	EX39	missense	Het	218	0.4
